# Neuroscientific evidence in the courtroom: a review

**DOI:** 10.1186/s41235-019-0179-y

**Published:** 2019-10-22

**Authors:** Darby Aono, Gideon Yaffe, Hedy Kober

**Affiliations:** 1 Yale College, Yale University, New Haven, CT USA; 2 Yale Law School, Yale University, New Haven, CT USA; 3 Departments of Psychiatry and Psychology, Yale University, New Haven, CT USA

**Keywords:** Neuroscience explanation, Legal decisions, Law, Neuroimages, Neuroimaging, Persuasion, Seductive allure

## Abstract

The use of neuroscience in the courtroom can be traced back to the early twentieth century. However, the use of neuroscientific evidence in criminal proceedings has increased significantly over the last two decades. This rapid increase has raised questions, among the media as well as the legal and scientific communities, regarding the effects that such evidence could have on legal decision makers. In this article, we first outline the history of neuroscientific evidence in courtrooms and then we provide a review of recent research investigating the effects of neuroscientific evidence on decision-making broadly, and on legal decisions specifically. In the latter case, we review studies that measure the effect of neuroscientific evidence (both imaging and nonimaging) on verdicts, sentencing recommendations, and beliefs of mock jurors and judges presented with a criminal case. Overall, the reviewed studies suggest mitigating effects of neuroscientific evidence on some legal decisions (e.g., the death penalty). Furthermore, factors such as mental disorder diagnoses and perceived dangerousness might moderate the mitigating effect of such evidence. Importantly, neuroscientific evidence that includes images of the brain does not appear to have an especially persuasive effect (compared with other neuroscientific evidence that does not include an image). Future directions for research are discussed, with a specific call for studies that vary defendant characteristics, the nature of the crime, and a juror’s perception of the defendant, in order to better understand the roles of moderating factors and cognitive mediators of persuasion.

## Significance

The increased use of neuroscientific evidence in criminal proceedings has led some to wonder what effects such evidence has on legal decision makers (e.g., jurors and judges) who may be unfamiliar with neuroscience. There is some concern that legal decision makers may be unduly influenced by testimony and images related to the defendant’s brain. This paper briefly reviews the history of neuroscientific evidence in the courtroom to provide context for its current use. It then reviews the current research examining the influence of neuroscientific evidence on legal decision makers and potential moderators of such effects. Our synthesis of the findings suggests that neuroscientific evidence has some mitigating effects on legal decisions, although neuroimaging-based evidence does not hold any special persuasive power. With this in mind, we provide recommendations for future research in this area. Our review and conclusions have implications for scientists, legal scholars, judges, and jurors, who could all benefit from understanding the influence of neuroscientific evidence on judgments in criminal cases.

## Introduction

Over the last four decades, the number of incarcerated Americans has increased by 500% (The Sentencing Project, 2018). In 2017, there were 1,097,083 arrests made in California alone (California Department of Justice, 2017), while an estimated total of 6,613,500 American citizens were on parole, probation, in jail, or in prison (Kaeble & Cowhig, 2018). Importantly, while incarceration rates have skyrocketed, the neuroscientific technology available for both criminal prosecution and defense has also increased at a rapid rate over the past few decades. From the advent of electroencephalography (EEG) in the 1930s to the first magnetic resonance imaging (MRI) scans performed on humans in the 1970s, the twentieth century saw great advances in neuroscience, and neuroimaging specifically. These tools not only gave scientists an inside view into the structure and function of the human brain, but they also allowed experts to better conceptualize the connection between the human brain and human behavior. This connection has become particularly evident, and relevant, in the courtroom.

The entrance of neuroscience to the courtroom has been featured in scientific and law review articles, as well as in numerous mainstream news articles, with titles ranging from “How criminal courts are putting brains—not people—on trial” (Gonzalez, 2017) to “Brain scans in the courts: prosecutor’s dream or civil rights nightmare?” (Gaines, 2018). Given how relatively new neuroscience is to the courtroom, there remain many open questions regarding its potential role. The purpose of this paper is to review the historical and current use of neuroscientific evidence by the legal system, as well as the current research investigating the effects of neuroscientific evidence on legal decision makers in criminal cases. Such a review is particularly timely in light of media, legal, and scientific concern over the potential biasing effect of such evidence (e.g., Choi, 2017; Davis, 2017).

## Neuroscience in the courtroom: a brief history

Neuroscience has been used in legal proceedings since the early twentieth century. Shen (2016) traces one of the earliest introductions of neuroscience into courtrooms to the 1940s, when EEG was first used in a case involving a defendant with epilepsy. At the time, EEG was used to shed light on diagnosing and treating epilepsy; some lawyers used this tool to argue against laws that denied rights to individuals with epilepsy, while others used it in an attempt to identify the neural markers of violence (Shen, 2016). By the mid-twentieth century, EEG had become such a common occurrence in epilepsy cases that psychiatrist and attorney Irwin Perr advised, “The lawyer interested in this subject must know some principles of electroencephalography—both in understanding and evaluating epilepsy and because of its frequent use as a tool in court cases” (Perr, 1958). Indeed, within a few decades of its invention, an understanding of EEG was recommended for attorneys, both for its probative value as well as its growing presence in the courtroom.

In 1981, John Hinckley’s attempted assassination of President Ronald Reagan led to one of the highest profile cases that utilized neuroscience in a criminal trial. Hinckley’s defense team introduced a computed tomography (CT) scan of his brain to help bolster its argument that he suffered from schizophrenia, and should therefore be found not guilty by reason of insanity (NGRI). Although the prosecution opposed the introduction of Hinckley’s CT scans as evidence, the district court judge ruled that the scans were admissible. Hinckley was ultimately found NGRI.

A decade later, a new form of neuroimaging made an appearance in *People v. Weinstein* (1992). Weinstein was charged with second-degree murder for strangling his wife and throwing her from the 12th floor of their Manhattan apartment, a charge he readily admitted to. His attorneys considered it suspicious that Weinstein would show so little remorse for his actions, and ordered positron emission tomography (PET) scans. At trial, Weinstein’s defense team presented his PET scans to support their claim that, due to an arachnoid cyst, his brain function was disrupted. Thus, they claimed that the defendant did not have the requisite mental state to be found criminally responsible. Weinstein was later allowed to plead guilty to the lesser charge of manslaughter.

Only a year after *Weinstein*, the rules governing the introduction of scientific evidence into federal trials changed significantly. In *Daubert v. Merrell Dow Pharmaceuticals, Inc.* (1993), two families sued Merrell Dow for their children’s birth defects, allegedly caused by the prenatal ingestion of a drug sold by the company. Although the district court granted summary judgment for Merrell Dow, the families appealed, and the case was eventually heard by the Supreme Court of the United States. Prior to *Daubert*, trial judges used the “*Frye* standard” to guide decisions on the admissibility of scientific testimony. The *Frye* standard dictated that, in order for testimony to be admitted to trial, the method by which the evidence was obtained must be “generally accepted” by the relevant scientific community. However, almost two decades before *Daubert*, Congress had passed the Federal Rules of Evidence (FRE), which offered a more liberal standard for allowing scientific testimony to enter trial. Rather than requiring “general acceptance” of the scientific technique for admissibility, the standard set by the FRE deemed such an assessment to be only one of a number to consider, along with whether or not the methodology is testable, whether it has been subjected to peer review, and its known or potential error rate. In *Daubert*, the Supreme Court replaced *Frye’s* supremacy in federal cases with the standard set forth by the FRE, which has come to be known as the “*Daubert* standard.” This opened the door for the more liberal use of scientific evidence in modern courtrooms. Specifically, the *Daubert* standard allowed scientific techniques and results that had not yet achieved general acceptance to appear in courtrooms. Thus, new imaging tools that were not yet widely used became admissible thanks to *Daubert*.

## Neuroscience in the modern courtroom

Along with its development in scientific contexts, the opportunity for neuroscience to be used as evidence in criminal trials has predictably increased since the turn of the century. Theoretically, neuroscientific evidence (broadly construed as any information related to the brain) can be used like any other type of evidence to establish or dispute any claim in a criminal case. It could be used, for example, to support or cast doubt on the testimony of an expert, to support or rebut a medical diagnosis, to corroborate a defendant’s testimony about his frame of mind at the time of the crime, to establish that a defendant’s conduct caused severe harm, or used demonstratively to help the judge or jury understand some other kind of evidence, and so on. In practice, the standard described in the prior section regulates the admission of such evidence in various courtrooms.

Meixner (2016) reviewed the use of neuroscientific evidence in criminal trials from 2005 to 2012 in the US, Canada, the Netherlands, England, and Wales. Summarizing prior findings, he reported that the use of neuroscientific evidence has increased at similar rates across all studied jurisdictions, with a sharp upwards slope from 2005 that levels off in around 2010. The absolute number of US cases involving neuroscientific evidence, however, has been significantly higher than the other jurisdictions.

In an analysis of US cases between 2005 and 2012, Farahany (2016) reported that 1585 judicial opinions from criminal cases mentioned the defense’s use of neuroscientific or genetic evidence. In 2012 alone, there were 250 judicial opinions written in which the criminal defendant argued (successfully or otherwise) that their “brain made them do it”. In another analysis, Farahany determined that neuroscientific and genetic evidence was introduced in 5% of all murder trials and 25% of all death penalty trials in 2012 (Farahany, 2016). In fact, 15% of the 1585 judicial opinions reviewed discussed such evidence specifically. It should be noted, however, that only a fraction of all criminal cases go to trial and end in guilty verdicts. Of these cases, only a fraction reach appellate court and subsequently generate written opinions. Therefore, this set of judicial opinions may not be representative of all cases, or even all cases that go to trial.

Denno (2015) provided a nuanced view of how neuroscience is used in criminal trials with her review of 553 criminal cases that presented neuroscientific evidence between 1992 and 2012. Two thirds (66.18%) of the cases began as death penalty cases, while 24.23% were cases in which either life or significant prison sentences (10+ years) were possible outcomes for the defendant. In nearly all cases, neuroscientific material was presented as mitigating evidence by the defense; in only 7% of cases was it presented as aggravating evidence by the prosecution. Although Denno did not quantify the claim, she reported that, across defense cases, neuroscientific evidence was often used to bolster a diagnosis that was already confirmed by a medical professional (Denno, 2015). Such diagnoses included substance use disorders, schizophrenia, depression, and organic brain damage (among others). However, in many cases neuroscientific evidence was used to suggest the existence of a “mental or behavioral” disorder that was not otherwise diagnosed. Interestingly, 63.29% of the reviewed cases specifically involved a form of neuroimaging evidence, including MRI, PET, and CT scans.

A particularly intriguing subset of the cases reviewed by Denno (2015) were those in which a defendant was convicted and subsequently argued that they had received “ineffective assistance of counsel” thanks to their attorney’s failure to introduce neuroscientific evidence. In *Strickland v. Washington* (1984), the Supreme Court ruled that in order for defendants to successfully appeal on account of ineffective assistance of counsel they must show that their attorneys performed below an “objective standard of reasonableness,” and that there was “a reasonable probability that, but for counsel’s unprofessional errors, the result of the proceeding would have been different.”

Such *Strickland* claims appeared in 53% of the cases reviewed by Denno (2015). Importantly, 87% of these *Strickland* claims included arguments that defense counsel presented insufficient neuroscientific evidence. Furthermore, 27.65% of the reported *Strickland* claims were successful (an extraordinarily high rate), with defense counsel’s inadequate use of neuroscientific evidence forming the basis of all but one successful claim. This success rate is especially striking given that *Strickland* claims are typically unsuccessful. For example, Benner (2009) reported a 4% success rate for all *Strickland* claims in California over a 10-year period. This difference in success rates likely stems from the types of cases reported by Denno (2015), namely, cases in which neuroscientific evidence was presented in the first place. Indeed, defendants who had a reason to introduce neuroscientific evidence in their original court cases (presumably due to neurological or mental abnormalities) may be more likely to successfully establish ineffective assistance of counsel compared with neurologically typical defendants. However, this high success rate may still suggest that the law is beginning to require defense lawyers to introduce neuroscientific evidence when it might prove valuable to the defendant’s case.

## Scientific investigations of courtroom neuroscience: definitions and scope

Following the above overview of the extent and nature of the role of neuroscience in criminal trials, we now focus on assessing the potential influence of such evidence on legal decision makers. In the US, criminal cases that do not end with plea bargains might be decided at a bench trial, over which the judge presides. However, most criminal cases that go to trial are decided by juries. In jury trials, jurors are responsible for both determining the facts of the case based on the presented evidence and for reaching a verdict (American Bar Association, 2018). Despite this responsibility, jurors are rarely experts in the types of evidence presented, nor are they trained in weighing evidence to arrive at legal conclusions. Therefore, juror response to neuroscientific evidence in which they have little, if any, expertise is of particular investigative interest.

Thus, here we focus on studies aiming to understand the effects of neuroscientific evidence on jurors in criminal trials. We define neuroscientific evidence as encompassing expert testimony related to brain structure or function and/or neuroimages presented as evidence. Expert testimony solely related to a mental disorder diagnosis, for example, was not considered neuroscientific evidence for the purposes of this paper, even if it was delivered by a neuroscience expert. Notably, the majority of studies in this field have been conducted on mock jurors (i.e., study participants who are asked to imagine themselves as part of a jury). Finally, the “effect” of neuroscientific evidence is measured via the legal judgments rendered by such mock jurors (e.g., guilty/not guilty, death penalty/life sentence).

To focus this review further, we used the framework suggested by Jones (2013) who helpfully outlined seven main categories for the application of neuroscience in the legal field: buttressing (the use of neuroscience as supporting evidence); detecting (the use of neuroscience to gain otherwise elusive insights, such as the extent of brain injuries); sorting (the use of neuroscience to categorize people into legal classifications, such as sane versus insane); challenging (the use of neuroscience to challenge an institutionalized assumption); intervening (the use of neuroscience to create and recommend interventions); explaining (the use of neuroscience to shed light on uncontested, yet not well understood phenomenon); and predicting (the use of neuroscience to help make predictions about people’s future behavior).

Accordingly, this review focuses on studies that examined the use of neuroscientific evidence as buttressing, detecting, or sorting devices. We chose to focus on these categories because there is both legal precedent and a relatively substantial body of research on the use of such neuroscientific evidence, while the other categories are in relatively earlier stages of examination. Therefore, in the reviewed studies, neuroscientific evidence is used to support an argument put forth by the criminal defense attorney, reveal brain damage relevant to the criminal case, or provide evidence for a diagnosable mental disorder. Finally, although technically within Jones’ category of detection, studies that focused on the use of neuroscientific evidence for lie detection purposes in criminal cases (e.g., McCabe, Castel, & Rhodes, 2011) were considered outside of the scope of this review.

## Scientific investigations of courtroom neuroscience: the empirical research literature

Empirical investigations of neuroscientific evidence in the courtroom were largely motivated by earlier studies exploring the effects of neuroscientific information on “regular” (nonlegal) judgments. In one of the pioneering studies on the broad persuasiveness of neuroscience outside of a courtroom context, Weisberg, Keil, Goodstein, Rawson, and Gray (2008) presented neuroscientifically naïve adult participants with brief descriptions of psychological phenomena (e.g., attentional blink), followed by more detailed explanations of the same phenomena. Importantly, the detailed explanations were either good or bad in quality, and either contained irrelevant neuroscientific information or no neuroscientific information at all, in a 2 (quality of argument) × 2 (presence of neuroscience information) design. Although the neuroscientific information was irrelevant, participants rated the scientific reasoning of bad explanations as more satisfying when it was included (there was no effect for good explanations). These findings were replicated in a second study with students in an introductory cognitive neuroscience class (Weisberg et al., 2008). Follow-up work showed that the effect that neuroscientific information renders explanations more satisfying did not depend on the length of the explanation or on neuroscientific jargon (Weisberg, Taylor, & Hopkins, 2015). Furthermore, this core finding (now termed “the seductive allure”) has since been replicated in much larger samples of neuroscientifically naïve participants (Michael, Newman, Vuorre, Cumming, & Garry, 2013). Together, these findings serve as the initial motivation for studies testing the effects of neuroscientific evidence on jurors.

While Weisberg et al. (2008) examined the influence of neuroscientific information, McCabe and Castel (2008) examined whether neuroimages held any power to bolster scientific arguments. Across two experiments, participants were presented with summaries of fictitious cognitive neuroscience studies (e.g., “watching TV is related to math ability”). Depending on the condition, the article was accompanied by a neuroimage, bar graph representing brain activity, topographical map of brain activation, or no image. Participants then rated whether “the scientific reasoning in the article made sense” on a four-point Likert scale. Overall, the results showed that participants presented with a neuroimage rated the scientific reasoning as making more sense compared with those who were presented with any other image, or no image. Thus, McCabe and Castel (2008) offered one of the first pieces of empirical evidence suggesting that neuroimages may hold a unique persuasive power over laypeople’s judgments, spurring several investigations into the effects of neuroimages on jurors.

However, these findings were later challenged by studies with similar designs that failed to replicate the persuasive influence of neuroimages. For example, Gruber and Dickerson (2012) compared the evaluations of participants on an article when it was presented with a neuroimage, an artistic rendering of a human head, an image from a movie, or no image. They found no differences across all conditions (Gruber & Dickerson, 2012). Similarly, Hook and Farah (2013) found that neuroimages had no effect on participants’ overall evaluation of or agreement with scientific articles when compared to stock photos or bar charts (they did not compare neuroimages to no images). The effects of neuroimages failed to replicate again in a series of studies using much larger samples (Michael et al., 2013).

Nevertheless, Weisberg and colleagues (Hopkins, Weisberg, & Taylor, 2016; Weisberg, Hopkins, & Taylor, 2018; Weisberg et al., 2008, 2015) and Michael et al. (2013) do provide evidence that laypeople’s evaluations of scientific claims may be affected by the mere presence of neuroscientific information, even when that information provides no additional value to the argument. These data have implications for everyday events (such as reading the news), as well as criminal trials, where the stakes of laypeople’s judgments are particularly high. To determine the extent of such implications, a number of studies have examined the effects of neuroscientific evidence on mock jurors. We review the extant research in an effort to answer the following questions: (1) Are legal judgments influenced by neuroscientific evidence (and, if so, what types of evidence)? (2) In which circumstances is neuroscientific evidence helpful and are there moderating factors? (3) Given the current state of the evidence, what might be productive avenues for future research?

It is important to note that this body of empirical work is methodologically varied. For example, some studies compare neuroscientific evidence accompanied by neuroimages with neuroscientific evidence without neuroimages (e.g., Schweitzer & Saks, 2011), while others only compare neuroscientific testimony with neuroimages to no neuroscientific testimony at all (e.g., Appelbaum, Scurich, & Raad, 2015). Additionally, although all reviewed studies asked participants to render a legal judgment on hearing the case, the types of legal judgments vary by study; for example, some studies asked for a guilty/not guilty verdict (e.g., Mowle, Edens, Clark, & Sörman, 2016), while others asked mock jurors to choose between guilty and NGRI (Schweitzer & Saks, 2011), or between the death penalty and a life sentence (Greene & Cahill, 2012). To provide a clear review of the current literature while accounting for wide methodological differences, we have organized the reviewed studies according to the types of evidence compared, separated into three sections: 1) neuroscientific expert testimony without neuroimages versus no neuroscientific testimony; 2) neuroscientific expert testimony with neuroimages versus no neuroscientific testimony; and 3) neuroscientific expert testimony with versus without neuroimages. Within each section, we organized studies by the type of legal judgment mock jurors were asked to render (e.g., guilty/not guilty, guilty/NGRI, sentence length; see Table 1).

**Table 1:** Summary of key variables for each reviewed study

**No expert neuroscientific testimony vs. Expert neuroscientific testimony without neuroimages**										
**Study**	**Number of Participants**	**Legal Judgment**	**Crime**	**Condition: No expert neuroscientific testimony**	**Condition: Expert neuroscientific testimony without neuroimages (Expert)**	**Expert Type**	**Additional Independent Variables**	**Additional Dependent Variables**	**Effect on Verdict**	**Effect on Sentence**
Saks et al., 2014 - Experiment 1	825	Death/Life sentence	First degree murder	No diagnosis or neuroscientific evidence	Two neuroscientists affirmed the mental disorder diagnosis based on fMRI scans of the defendant’s brain	Neuroscientists	Diagnosis: Schizophrenia vs. psychopathy vs. healthy	Responsibility, dangerousness	N/A	Yes: Reduced death sentences, only for defendants with Schizophrenia
Greene & Cahill, 2012	208	Death/Life sentence	First degree murder	Neuropsychologist testified to defendant’s psychosis diagnosis	Neuropsychologist testified to psychosis diagnosis and reported cognitive deficits suggesting frontal brain damage	Neuropsychologist	Defendant dangerousness: low vs. high risk	Responsibility, self-control, dangerousness, influence of expert testimony	N/A	Yes: Reduced death sentences, only for high risk defendants
Schweitzer et al., 2011 - Experiment 1	237	Verdict (first-degree/second-degree/manslaughter/not guilty) + Sentence	Armed robbery + homicide	Defense attorney claimed defendant suffered from a neurological defect preventing him from forming the requisite intent.	Neuroscientist claimed that the defendant suffered from structural frontal lobe damage, preventing him from being able to premeditate and deliberate about, or form the intent to be guilty of, first or second degree murder. He was also prone to losing control and becoming enraged.	Neuroscientist	N/A	N/A	No: No change in verdict	No: No change in sentence
Schweitzer et al., 2011 - Experiment 2	294	Verdict (guilty/not guilty) + Sentence	Robbery + assault	Defense attorney claimed defendant suffered from a neurological defect preventing him from forming the requisite intent.	Neuroscientist claimed that the defendant suffered from structural frontal lobe damage, preventing him from being able to premeditate and deliberate about, or form the intent to be guilty of, first or second degree murder. He was also prone to losing control and becoming enraged.	Neuroscientist	N/A	Responsibility, self-control	No: No change in verdict	No: No change in sentence
Schweitzer et al., 2011 - Experiment 3	512	Verdict (simple assault/aggravated assault/not guilty) + Sentence	Assault	Defense attorney claimed defendant suffered from a neurological defect preventing him from forming the requisite intent.	Neuroscientist claimed that the defendant suffered from structural or functional frontal lobe damage, preventing him from being able to premeditate, or form the intent to be guilty of, first or second degree murder. He was also prone to losing control and becoming enraged.	Neuroscientist	N/A	Responsibility, self-control	No: No change in verdict	No: No change in sentence
Schweitzer et al., 2011 - Experiment 4	433	Verdict (simple assault/aggravated assault/not guilty) + Sentence	Assault	Defense attorney claimed defendant suffered from a neurological defect preventing him from forming the requisite intent.	Neuroscientist claimed that the defendant suffered from structural frontal lobe damage, preventing him from being able to premeditate and deliberate about, or form the intent to be guilty of, first or second degree murder. He was also prone to losing control and becoming enraged.	Neuroscientist	N/A	Responsibility, self-control	No: No change in verdict	No: No change in sentence
Schweitzer et al., 2011 - Meta-analysis	1374	Meta-analysis	Meta-analysis	Meta-analysis	Meta-analysis	Meta-analysis	N/A	Responsibility, self-control	No: No change in verdict	No: No change in sentence
Mowle et al., 2016	419	Guilty/Not guilty + Sentence	Robbery + assault	Psychologist testified to defendant’s mental disorder and traumatic brain injury (Condition 2).	Psychologist testified to defendant’s diagnosis and damage to prefrontal cortex, predisposing him to impulsivity (Condition 3).	Psychologist	Diagnosis: Schizophrenia vs. psychopathy	N/A	No: No change in verdict	No: No change in sentence
Allen et al., 2019	330	Sentence	Sexual assault	Psychologists diagnosed defendant with an impulse control disorder	Neurologists had located a large tumor in the “impulse control” region of the defendant’s brain	Neurologists	Treatment/dangerousness: treated and low risk of future dangerousness vs. untreatable and high risk of future dangerousness	Responsibility, self-control, importance of expert testimony	N/A	Yes: Reduced length of prison sentences
LaDuke et al., 2018	896	Sentence	Burglary + aggravated assault	Facts of case (mock police report of the crime, defendant statement, defendant plea); allusion to family and friend statement in support of defendant.	[VIDEO] Psychologist described the defendant’s neurological abnormalities, based on MRI/fMRI scans, and concluded that the defendant posed a high risk for future violence.	Psychologist	Structural vs. functional neuroimaging	Culpability, dangerousness, influence of expert testimony	N/A	No: No change in sentence
Marshall et al., 2017 - Experiment 2	400	Sentence	Murder	Psychiatrist discussed psychological interview methods for psychopaths, testified that the defendant exhibited a lack of impulse control and feelings of guiltlessness, explained characteristics of psychopaths.	Neuroscientist discussed fMRI research and psychopathy measurement techniques for psychopaths, testified that the defendant exhibited underactivation in amygdala and orbitofrontal cortex, explained characteristics of psychopaths.	Neuroscientist	N/A	Self-control, dangerousness, influence of expert testimony	N/A	No: No change in sentence
**No expert neuroscientific testimony vs. Expert neuroscientific testimony with neuroimages**										
**Study**	**Number of Participants**	**Legal Judgment**	**Crime**	**Condition: No expert neuroscientific testimony**	**Condition: Expert neuroscientific testimony with neuroimages (Expert+Neuroimage)**	**Expert Type**	**Additional Independent Variables**	**Additional Dependent Variables**	**Effect on Verdict**	**Effect on Sentence**
Saks et al., 2014 - Experiment 1	825	Death/Life sentence	First-degree murder	No diagnosis or neuroscientific evidence	Expert + fMRI	Neuroscientists	Diagnosis: Schizophrenia vs. psychopathy vs. healthy	Responsibility, dangerousness	N/A	No: No change in death sentences
Greene & Cahill, 2012	208	Death/Life sentence	First-degree murder	Neuropsychologist testified to defendant’s psychosis diagnosis	Expert + MRI and PET with descriptions of behavioral implications.	Neuropsychologist	Defendant dangerousness: low vs. high risk	Responsibility, self-control, dangerousness, influence of expert testimony	N/A	Yes: Reduced death sentences, only for high risk defendants
Appelbaum et al., 2015 - Experiment 3	763	Death/Life sentence	First-degree murder	Defense attorney claimed that the defendant’s act was impulsive	Psychiatrist testified that neuroimage showed a brain abnormality which led the defendant to act impulsively. Presented MRI.	Psychiatrist	Crime heinousness: low vs. high	Dangerousness, self-control	N/A	Yes: Reduced death sentences
Schweitzer et al., 2011 - Experiment 1	237	Verdict (first-degree/second-degree/manslaughter/not guilty) + Sentence	Armed robbery + homicide	Defense attorney claimed defendant suffered from a neurological defect preventing him from forming the requisite intent.	Expert + fMRI	Neuroscientist	N/A	N/A	No: No change in verdict	No: No change in sentence
Schweitzer et al., 2011 - Experiment 2	294	Verdict (guilty/not guilty) + Sentence	Robbery + assault	Defense attorney claimed defendant suffered from a neurological defect preventing him from forming the requisite intent.	Expert + fMRI	Neuroscientist	N/A	Responsibility, self-control	No: No change in verdict	No: No change in sentence
Schweitzer et al., 2011 - Experiment 3	512	Verdict (simple assault/aggravated assault/not guilty) + Sentence	Assault	Defense attorney claimed defendant suffered from a neurological defect preventing him from forming the requisite intent.	Expert + MRI and fMRI	Neuroscientist	N/A	Responsibility, self-control	No: No change in verdict	No: No change in sentence
Schweitzer et al., 2011 - Experiment 4	433	Verdict (simple assault/aggravated assault/not guilty) + Sentence	Assault	Defense attorney claimed defendant suffered from a neurological defect preventing him from forming the requisite intent.	Expert + MRI	Neuroscientist	N/A	Responsibility, self-control	Yes: Reduced verdict severity	No: No change in sentence
Schweitzer et al., 2011 - Meta-analysis	1374	Meta-analysis	Meta-analysis	Meta-analysis	Meta-analysis	Meta-analysis	N/A	Responsibility, self-control	Yes: Reduced guilty verdicts	No: No change in sentence
Mowle et al., 2016	419	Guilty/Not guilty + Sentence	Robbery + assault	Psychologist testified to defendant’s mental disorder and traumatic brain injury (Condition 2).	Expert + Brain scan	Psychologist	Diagnosis: Schizophrenia vs. psychopathy	N/A	No: No change in verdict	No: No change in sentence
LaDuke et al., 2018	896	Sentence	Burglary + aggravated assault	Facts of case (mock police report of the crime, defendant statement, defendant plea); allusion to family and friend statement in support of defendant.	Expert + MRI/fMRI (two neuroimage conditions)	Psychologist	Structural vs. functional neuroimaging	Culpability, dangerousness, influence of expert testimony	N/A	No: No change in sentence
Appelbaum et al., 2015 - Experiment 1	960	Sentence	Murder	Defense attorney claimed that the defendant’s act was impulsive	Psychiatrist testified that neuroimage showed a brain abnormality which predisposed the defendant to impulsivity and violence. Presented MRI.	Psychiatrist	Crime heinousness: low vs. high	Dangerousness, self-control	N/A	No: No change in sentence
Schweitzer & Saks, 2011	1170	Guilty/NGRI or GBMI	Assault	Defense attorney argued that the defendant suffered from a mental disorder causing him to act irrationally and uncontrollably. Family testified to neglect and abuse of defendant as a child.	Neurologist testified to MRI showing physical damage to frontal lobe, which could cause defendant to lose control over actions. Presented MRI. Second expert emphasized important of frontal lobe.	Neurologist	N/A	Self-control	Yes: Reduced guilty verdicts	N/A
Gurley & Marcus, 2008	394	Guilty/NGRI	Murder	Psychologist and psychiatrist testified to defendant’s diagnosis	Psychologist and psychiatrist testified to diagnosis and presented 4 MRI scans showing prefrontal cortex damage, which likely contributed to difficulty with impulse control.	Psychologist and psychiatrist	Diagnosis: Psychosis (schizophrenia) vs. psychopathy	Influence of expert testimony	Yes: Reduced guilty verdicts	N/A
**Expert neuroscientific testimony without neuroimages vs. Expert neuroscientific testimony with neuroimages**										
**Study**	**Number of Participants**	**Legal Judgment**	**Crime**	**Condition: Expert neuroscientific testimony without neuroimages (Expert)**	**Condition: Expert neuroscientific testimony with neuroimages (Expert+Neuroimage)**	**Expert Type**	**Additional Independent Variables**	**Additional Dependent Variables**	**Effect on Verdict**	**Effect on Sentence**
Saks et al., 2014 - Experiment 1	825	Death/Life sentence	First degree murder	Two neuroscientists affirmed the mental disorder diagnosis based on fMRI scans of the defendant’s brain	Expert + fMRI	Neuroscientists	Diagnosis: Schizophrenia vs. psychopathy vs. healthy	Responsibility, dangerousness	N/A	No: No change in death sentences
Greene & Cahill, 2012	208	Death/Life sentence	First-degree murder	Neuropsychologist testified to psychosis diagnosis and reported cognitive deficits suggesting frontal brain damage	Expert + MRI and PET with descriptions of behavioral implications.	Neuropsychologist	Defendant dangerousness: low vs. high risk	Responsibility, self-control, dangerousness, influence of expert testimony	N/A	No: No change in death sentences
Schweitzer et al., 2011 - Experiment 1	237	Verdict (first-degree/second-degree/manslaughter/not guilty) + Sentence	Armed robbery + homicide	Neuroscientist claimed that the defendant suffered from structural frontal lobe damage, preventing him from being able to premeditate and deliberate about, or form the intent to be guilty of, first or second degree murder. He was also prone to losing control and becoming enraged.	Expert + fMRI	Neuroscientist	N/A	N/A	No: No change in verdict	No: No change in sentence
Schweitzer et al., 2011 - Experiment 2	294	Verdict (guilty/not guilty) + Sentence	Robbery + assault	Neuroscientist claimed that the defendant suffered from structural frontal lobe damage, preventing him from being able to premeditate and deliberate about, or form the intent to be guilty of, first or second degree murder. He was also prone to losing control and becoming enraged.	Expert + fMRI	Neuroscientist	N/A	Responsibility, self-control	No: No change in verdict	No: No change in sentence
Schweitzer et al., 2011 - Experiment 3	512	Verdict (simple assault/aggravated assault/not guilty) + Sentence	Assault	Neuroscientist claimed that the defendant suffered from structural or functional frontal lobe damage, preventing him from being able to premeditate, or form the intent to be guilty of, first or second degree murder. He was also prone to losing control and becoming enraged.	Expert + MRI and fMRI	Neuroscientist	N/A	Responsibility, self-control	No: No change in verdict	No: No change in sentence
Schweitzer et al., 2011 - Experiment 4	433	Verdict (simple assault/aggravated assault/not guilty) + Sentence	Assault	Neuroscientist claimed that the defendant suffered from structural frontal lobe damage, preventing him from being able to premeditate and deliberate about, or form the intent to be guilty of, first or second degree murder. He was also prone to losing control and becoming enraged.	Expert + MRI	Neuroscientist	N/A	Responsibility, self-control	No: No change in verdict	No: No change in sentence
Schweitzer et al., 2011 - Meta-analysis	1374	Meta-analysis	Meta-analysis	Meta-analysis	Meta-analysis	Meta-analysis	N/A	Responsibility, self-control	No: No change in verdict	No: No change in sentence
Mowle et al., 2016	419	Guilty/Not guilty + Sentence	Robbery + assault	Psychologist testified to defendant’s diagnosis and damage to prefrontal cortex, predisposing him to impulsivity.	Expert + Brain scan	Psychologist	N/A	N/A	No: No change in verdict	No: No change in sentence
LaDuke et al., 2018	896	Sentence	Burglary + aggravated assault	[VIDEO] Psychologist described the defendant’s neurological abnormalities, based on MRI/fMRI scans, and concluded that the defendant posed a high risk for future violence.	Expert + MRI/fMRI (two neuroimage conditions)	Psychologist	Structural vs. functional neuroimaging	Culpability, dangerousness, influence of expert testimony	N/A	No: No change in sentence
Marshall et al., 2017 - Experiment 1	758	Sentence	Murder	Neuroscientist discussed fMRI research and psychopathy measurement techniques for psychopaths, testified that the defendant exhibited underactivation in amygdala and orbitofrontal cortex, explained characteristics of psychopaths.	Expert + fMRI	Neuroscientist	N/A	Self-control, dangerousness, influenced by expert testimony	N/A	No: No change in sentence
Schweitzer & Saks, 2011	1170	Guilty/NGRI or GBMI	Assault	Neurologist testified to MRI showing physical damage to frontal lobe, which could cause defendant to lose control over actions. Second expert emphasized important of frontal lobe.	Expert + MRI	Neurologist	N/A	Self-control	No: No change in verdict	N/A
Baker et al., 2013	73	Guilty/Not guilty	Assault	Neurologist testified that an MRI revealed frontal lobe damage and that such damage could impair impulse control. Presented **bar graph** comparing normal brain activity to defendant’s brain activity.	Neurologist testified that an MRI revealed frontal lobe damage and that such damage could impair impulse control. Presented MRI scans of normal brain and defendant’s brain.	Neurologist	N/A	Dangerousness	No: No change in verdict	No: No change in degree of punishment

*N/A* Data were not available or the variable was not used, *fMRI* Functional magnetic resonance imaging, *MRI* Magnetic resonance imaging, *NGRI* Not guilty by reason of insanity, *GBMI* Guilty but mentally ill

## Does neuroscientific expert testimony affect juror decisions?

Several studies examined the effects of neuroscientific expert testimony (without neuroimages) by comparing mock juror decision-making in its presence versus its absence (Table 1). For example, Saks, Schweitzer, Aharoni, and Kiehl (2014) presented mock jurors with a defendant who had been convicted of first-degree murder. The mock jurors were asked to decide whether to sentence the defendant to death or to life in prison. The defendant was described as healthy, diagnosed with schizophrenia, or diagnosed with psychopathy. The authors note that they chose these disorders as they both commonly coincide with behavioral disinhibition (Saks et al., 2014). In the neuroscientific testimony conditions, mock jurors were told that the defense presented expert testimony from two neuroscientists who, having examined functional MRI (fMRI) scans of the defendant’s brain, affirmed the mental disorder diagnosis (or lack thereof). In the control condition, mock jurors were presented with the same case, but were not presented with any expert testimony supporting the diagnosis.

Saks et al. (2014) found a mitigating effect of neuroscientific testimony on death penalty rates. This effect was moderated by diagnosis; defendants who had been diagnosed with schizophrenia were sentenced to death less often when mock jurors were presented with neuroscientific testimony. This effect did not hold for defendants who had been diagnosed with psychopathy or defendants who had been described as healthy; neuroscientific testimony had no mitigating effects on death sentences for these defendants. Thus, this study suggests that neuroscientific expert testimony can have a mitigating effect on death penalty sentences under some conditions, namely, for defendants diagnosed with schizophrenia.

Greene and Cahill (2012) also compared the effects of neuroscientific testimony on death sentences for a defendant convicted of first-degree murder of his cellmate. In their study, however, the defendant was diagnosed with psychosis in all conditions. In the neuroscientific expert testimony condition, a psychologist testified to the defendant’s diagnosis of psychosis. The psychologist summarized neuropsychological tests that revealed cognitive deficiencies (e.g., lack of behavioral control, poor impulse control), and testified that these results suggest damage to the frontal area of the defendant’s brain. In the control condition, the psychologist only testified that the defendant suffered from psychosis, and that such a mental disorder would likely influence his behavior (i.e., the control condition neither referenced neuropsychological tests nor suggested brain damage). Across all conditions, the researchers varied the testimony of the prosecution’s expert witness, who testified that the defendant posed either a high or low risk of future dangerousness.

The authors reported that the defendant’s risk of future dangerousness moderated the effect of neuroscientific testimony on death sentences. Specifically, when the defendant was described as low-risk, the neuroscientific testimony did not affect sentences. However, neuroscientific testimony was significantly mitigating when the defendant was described as high-risk. In fact, mock jurors were 12 times less likely to sentence the defendant to death in the neuroscientific testimony condition compared with the control condition. Again, results from this study suggest neuroscientific expert testimony can have a mitigating effect on death penalty sentences under some conditions, in this case when the defendant is characterized as being at high risk of future dangerousness.

Testing the effects of neuroscientific testimony on guilty/not guilty verdicts, Schweitzer et al. (2011) performed four experiments with similar designs, and then quantitatively summarized their results in a meta-analysis. In each of the four experiments, mock jurors were presented with a case in which a defendant was charged with a violent crime. The defense attorney argued that the defendant suffered from a “neurological defect” preventing him from being able to form the requisite intention to harm. In the neuroscientific expert testimony conditions, a neuroscience expert testified that a defect in the defendant’s frontal lobe, discovered via (an unpresented) brain scan, prevented him from being able to form the intent necessary to be found guilty of murder. In the control conditions, there was no neuroscientific testimony supporting the defense’s claims. Mock jurors were then asked to choose a verdict, which, in three of the experiments, spanned multiple degrees of guilt (e.g., first-degree murder, second-degree murder, manslaughter). If the mock juror returned a guilty verdict, they were also asked to recommend a sentence.

Across all four experiments, the presence of neuroscientific testimony regarding the defendant’s defective frontal lobe failed to mitigate either verdicts or sentences. Their meta-analysis similarly found no effects of neuroscientific testimony on these outcomes, suggesting that neuroscientific testimony does not affect guilty verdicts or sentencing decisions (Schweitzer et al., 2011).

Mowle et al. (2016) tested the effects of neuroscientific expert testimony on simple guilty/not guilty verdicts. Mock jurors were told that the defendant was being charged with robbing a woman and slashing her face with a knife. Across all conditions, a psychologist described the defendant as either “a psychopath” or “a schizophrenic” and described symptoms of the disorder. In the control condition, a psychologist testified that the defendant had suffered a traumatic brain injury 6 months prior in an automobile accident. In the neuroscientific evidence condition, the psychologist also testified that the defendant had significant damage to his prefrontal cortex and that individuals with such damage are impulsive and less likely to control their actions. The mock jurors were then asked to return a verdict and, if they deemed the defendant guilty, a recommended sentence. Results showed no effects of neuroscientific evidence on verdict or sentence length. Diagnosis type did not moderate the effect of neuroscientific evidence on verdict or sentence length.

More recently, Allen, Vold, Felsen, Blumenthal-Barby, and Aharoni (2019) tested the effects of neuroscientific expert testimony on prison sentences for a defendant found guilty of sexually assaulting a woman. Notably, participants in this study were instructed to act as mock judges, not jurors. In the neuroscientific expert testimony conditions, participants were told that neurologists had conducted “MRI scans of the defendant’s brain” and concluded that the defendant had a “large tumor in a part of the brain involved in impulse control,” which could explain his impulsive criminal behavior. In the control condition, participants were told that psychologists had conducted “a series of clinical interviews with the defendant” and concluded that the defendant had an impulse control disorder, which could explain his behavior (note that these conditions differ not only in neuroscience content, but also in whether the defendant has a tumor versus an impulse control disorder). Under both conditions, half of the participants heard that the defendant’s affliction had been treated and he was therefore at low risk of future dangerousness, while the rest heard that it was untreatable and he was thus at high risk of future dangerousness. Participants were additionally asked about their beliefs regarding the defendant, including his moral responsibility, moral wrongness, blameworthiness, desert of punishment, self-control, and free will.

Allen et al. (2019) found that participants in the neuroscientific expert condition recommended significantly shorter sentences than those in the control condition. Importantly, further analyses revealed that beliefs about the defendant (e.g., his moral responsibility, blameworthiness, self-control, free will) fully accounted for the mitigating effect of the neuroscientific evidence. Testimony regarding the defendant’s treatment and risk of future dangerousness also had a significant effect on sentences; defendants who were successfully treated and were at low risk of future dangerousness received shorter prison sentences than those whose treatment was unsuccessful and therefore posed a higher risk of future dangerousness. However, the researchers did not find any interaction between expert testimony and treatment/risk of future dangerousness.

All the studies reviewed thus far have presented participants with neuroscientific expert testimony in written form. However, this format is actually unrepresentative of the typical trial experience, which generally involves in-person testimony. LaDuke, Locklair, and Heilbrun (2018) attempted to mimic juror experience by presenting mock jurors with video expert testimony. In their study, the defendant had been found guilty of burglary and aggravated assault and was now awaiting sentencing. In the neuroscientific expert testimony conditions, mock jurors were shown a video of an expert psychologist (interestingly, the expert was not presented as either a witness for the defense or for the prosecution). The expert described the defendant’s brain scans which suggested neurological abnormalities, as well as the behavioral implications of such abnormalities. In one expert condition, the expert referenced a structural MRI scan, and in the other, he cited an fMRI scan. In both conditions, the expert concluded by saying that, in his professional opinion, the defendant posed a high risk for future dangerousness. In the control condition, mock jurors were only presented with the facts of the case; they did not see any expert testimony. LaDuke et al. (2018) found no difference in sentences between conditions, and thus no mitigating effect of neuroscientific evidence. Finally, Marshall, Lilienfeld, Mayberg, and Clark (2017) compared neuroscientific expert testimony with a psychiatric expert testimony in a murder case (see Table 1 for experiment details). The researchers found no difference in recommended prison sentences between the neuroscientific and psychiatric expert conditions.

Taken together, the data suggest that neuroscientific expert testimony may be mitigating under certain circumstances; specifically, it may lead mock jurors to forgo the death penalty (i.e., Greene & Cahill, 2012; Saks et al., 2014). However, even in death penalty cases, such evidence was mitigating for only a subset of mock jurors. For example, Saks et al. (2014) showed that neuroscientific evidence was mitigating for defendants diagnosed with schizophrenia, but not for those diagnosed with psychopathy. Greene and Cahill (2012) showed that, across defendants with psychosis, neuroscientific testimony was mitigating only when the defendant was described as posing a high risk of future dangerousness. Greene and Cahill (2012) specifically hypothesized that the psychosis diagnosis in conjunction with the low-risk danger assessment already substantially mitigated the defendant’s death sentence, making any additional mitigating testimony superfluous. By contrast, high-risk defendants may benefit more from expert neuroscientific testimony.

Importantly, across studies, neuroscientific testimony does not appear to have a consistently mitigating effect on guilty/not guilty decisions (Mowle et al., 2016; Schweitzer et al., 2011), or on sentencing (LaDuke et al., 2018; Marshall et al., 2017; Mowle et al., 2016; Schweitzer et al., 2011). Notably, this was the case even for defendants who posed a high risk of future dangerousness (LaDuke et al., 2018) and those with diagnoses of mental illness (Marshall et al., 2017; Mowle et al., 2016). Indeed, only a single study (Allen et al., 2019) found a mitigating effect of neuroscientific testimony on prison sentences. Overall, one plausible explanation is that the effects of neuroscientific testimony are strong enough to prevent a death sentence for some defendants (or reduce the prison sentence in one study), but too weak to introduce reasonable doubt of guilt.

## Does neuroscientific expert testimony accompanied by neuroimages affect juror decisions?

Several studies examined the effects of neuroscientific evidence in the form of an expert testimony accompanied by a neuroimage, and compared such conditions with those in which no neuroscientific evidence was provided (i.e., no neuroscientific testimony or image; Table 1). Such comparisons do not isolate the effects of neuroimages on mock jurors, but rather test the combined effects of these two forms of neuroscientific evidence. We review these studies in the present section and discuss their results further in the “General discussion” section.

Appelbaum et al. (2015) performed two experiments. In the first, the defendant was described as having stabbed the victim to death, and the defense argued for a shorter prison sentence; in the second, the defendant was described as having shot and killed a police officer, and the defense argued for a life sentence over the death penalty. In the control condition in both cases, the defense attorney claimed that the defendant’s act was impulsive. In the combined expert+neuroimage conditions, a psychiatrist presented an MRI scan of the defendant’s brain and testified that it showed functional abnormalities predisposing him to impulsivity and violent behavior. Additionally, the crime was described as being of either low or high heinousness (e.g., one stab wound versus 17 stab wounds). Appelbaum et al. (2015) found no effect of neuroscientific evidence on the length of sentence in the first experiment. Such evidence did, however, reduce the death penalty rate in the second experiment compared with the control condition. The heinousness of the crime did not moderate this effect.

Greene and Cahill (2012) also tested the expert+neuroimage combination on death sentences (the study is described in full in the prior section, as well as in Table 1). Across all conditions, the expert described the defendant as “psychotic.” In the expert+neuroimage condition, a psychologist described the defendant’s neuropsychological tests, images of his damaged brain, and provided testimony regarding the likely behavioral consequences of such brain damage. In the control condition, the psychologist only testified that the defendant’s psychosis would likely influence his behavior. The defendant’s risk of future dangerousness also varied by condition (high versus low risk). The results showed that the defendant’s dangerousness moderated the mitigating effects of the evidence; mock jurors in the expert+neuroimage condition were 22 times less likely to sentence a high-risk defendant to death than mock jurors in the control condition. Low-risk defendants, however, were sentenced to death at the same rate across both conditions.

Conversely, Saks et al. (2014) found no change in death sentence rates following expert testimony accompanied by neuroimages (see prior section and Table 1). In the expert+neuroimage condition, the defense presented expert testimony from two neuroscientists who presented fMRIs of the defendant’s brain and affirmed his mental disorder diagnosis (schizophrenia, psychopathy, or healthy). In the control condition, mock jurors were not presented with any expert testimony or neuroimages supporting the diagnosis. The null effect of expert+neuroimage in this study is surprising given that, in the same study, expert testimony without neuroimages was mitigating for defendants diagnosed with schizophrenia (Saks et al., 2014). However, because this disparity (i.e., mitigating effects without brain images, but no effect with brain images) has not been replicated by any other study to our knowledge, we will not interpret it further at this time.

Several other studies have examined the effects of expert+neuroimage conditions on guilty/not guilty verdicts and sentences. These include the four experiments (and their associated meta-analysis) by Schweitzer and colleagues (Schweitzer et al., 2011), which tested the effects of expert+neuroimage evidence on guilty verdicts and sentences (see above, and Table 1). In the combined expert+neuroimage conditions, a neuroscientist presented the defendant’s fMRI scans and claimed that his frontal lobe was defective in such a way that he could not have formed the necessary intent required for conviction. Mock jurors in the control condition only read the defense’s argument.

Again, Schweitzer et al. (2011) found no effect of expert+neuroimage on verdict or sentence in the first three experiments. The fourth experiment, however, removed one aspect of the case summary—in the first three experiments, part of the defense included family testimony that the defendant was physically abused as a child. Without this family testimony, mock jurors in the expert+neuroimage condition returned more lenient verdicts than those in the control condition (e.g., simple versus aggravated assault). There was no effect on sentences, however. In the final meta-analysis, the researchers found a significant 12% reduction in guilty verdicts between the expert+neuroimage conditions and the control conditions, but no difference in sentences.

Mowle et al. (2016; see above and Table 1) presented all mock jurors with expert testimony from a psychologist describing the defendant’s diagnosis (psychopathy or schizophrenia) and his history of traumatic brain injury. In the combined expert+neuroimage condition, the psychologist also testified that the defendant had significant damage to his prefrontal cortex, and that individuals with such damage are impulsive and less likely to control their actions than someone with an undamaged brain. This testimony was accompanied by an image of the defendant’s brain, with the brain damage highlighted. Results showed no effects of the expert testimony on verdict or sentence length. Similarly, LaDuke et al. (2018; described above and in Table 1) found no effect of the of the combined expert+neuroimage condition on sentence length for a defendant convicted of burglary and aggravated assault. These null findings held for both the MRI and the fMRI conditions.

In addition, two studies examined the effects of neuroscientific evidence on NGRI verdicts. For example, Schweitzer and Saks (2011; see above and Table 1) described expert+neuroimage conditions in which a neurologist testified that the defendant’s brain damage (presented in an MRI) could cause him to lose control over his actions. In the control condition, there was no expert testimony supporting the defense’s claim of a mental disorder. Depending on the condition, mock jurors were instructed to return a verdict of guilty versus guilty but mentally ill (GBMI) or guilty versus NGRI. Results showed that mock jurors in the expert+neuroimage conditions were more likely to render NGRI/GBMI verdicts than those in the control condition. Furthermore, the mitigating effect of neuroscientific evidence on verdicts was mediated by the amount of control the mock juror believed the defendant had over his actions.

Gurley and Marcus (2008) also tested the effects of expert+neuroimage on NGRI verdicts. In their study, all mock jurors were presented with a murder committed by the defendant and expert testimony that the defendant suffered from a mental disorder (psychosis or psychopathy). In the expert+neuroimage conditions, a psychologist and a psychiatrist supported the diagnosis with MRI scans showing extensive damage to the prefrontal cortex, and described the relationship between such prefrontal damage and impulse control problems. In the control condition, there was no neuroscientific evidence presented in support of the diagnosis. Importantly, results showed that mock jurors in the expert+neuroimage conditions were significantly more likely to find the defendant NGRI than those who had not been given any neuroscientific evidence in support of the diagnosis (Gurley & Marcus, 2008). Interestingly, diagnosis did not moderate the effect of expert+neuroimages on the verdict as it did for expert testimony in other studies (e.g., Saks et al., 2014). However, mock jurors who rendered the NGRI verdict reported that they were more influenced by the expert testimony compared with those who rendered the guilty verdict.

Taken together, the data in this section suggest that, similar to expert testimony alone, neuroscientific expert testimony accompanied by neuroimages may be mitigating under certain circumstances. Specifically, it led mock jurors to forgo the death penalty in one study (Appelbaum et al., 2015) although not another (Saks et al., 2014). In another study, such evidence was mitigating for a subset of defendants described as posing a high risk of future dangerousness (Greene & Cahill, 2012). Importantly, expert+neuroimage conditions mitigated NGRI/GBMI verdicts compared with control conditions (Gurley & Marcus, 2008; Schweitzer & Saks, 2011), a verdict type that was not tested in any study with expert testimony alone. Notably, the defendant’s mental disorder diagnosis did not appear to moderate the effect of the expert+neuroimage on NGRI verdicts (Gurley & Marcus, 2008).

Furthermore, unlike the effects of expert testimony alone, the combination of expert+neuroimage also had a mitigating effect on guilty/not guilty verdicts in some studies (Schweitzer et al., 2011), while several other studies reported no effects (Mowle et al., 2016; Schweitzer et al., 2011). Similar to expert testimony alone, none of the studies reported effects of expert+neuroimage on sentence length (Appelbaum et al., 2015; LaDuke et al., 2018; Mowle et al., 2016; Schweitzer et al., 2011).

## Do neuroimages affect juror decisions above and beyond neuroscientific expert testimony?

Several of the aforementioned studies attempted to isolate the effects of neuroimages on mock jurors by comparing expert conditions to expert+neuroimage conditions. In other words, they asked whether neuroimages enhance neuroscientific expert testimony (for details, see Table 1). Perhaps unremarkably, given the failure to replicate the findings of McCabe and Castel (2008), none of the studies found a significant mitigating effect of neuroimages above and beyond that of the expert testimony. Specifically, both Schweitzer et al. (2011) and Mowle et al. (2016) found no differences in guilty/not guilty verdicts, even when mock jurors were offered a range of possible guilty verdicts (e.g., first-degree murder, second-degree murder, manslaughter). Similarly, no differences were reported in recommended sentence lengths when neuroimages were introduced with the xpert testimony, compared with the same expert testimony without neuroimages (LaDuke et al., 2018; Marshall et al., 2017; Mowle et al., 2016; Schweitzer et al., 2011).

Finally, although expert testimony (with and without brain images) was mitigating in several studies describing death penalty and NGRI cases, similar effects were not found for neuroimages (Greene & Cahill, 2012; Saks et al., 2014; Schweitzer & Saks, 2011). One additional study compared a neuroimage with a bar graph (both accompanied by neuroscientific expert testimony) and reported no differences (Baker, Schweitzer, Risko, & Ware, 2013). Together, these null findings suggest that, while neuroscientific expert testimony with and without neuroimages may lead to more lenient outcomes for defendants, neuroimages themselves hold very little, if any, mitigating power.

## General discussion

As multiple analyses have shown, the use of neuroscientific evidence in criminal proceedings has increased tremendously in the US within the last two decades (Meixner, 2016). This trend holds true for both neuroscientific evidence in general, and neuroimage-based evidence specifically (Denno, 2015). Indicative of an even greater systemic change, defendants have begun making, and winning, *Strickland* claims on the basis that their attorney neglected to present neuroscientific evidence in their defense. These claims argue that, had neuroscientific evidence been presented, the case's outcome would likely have been different (Denno, 2015).

The growing prevalence of neuroscientific evidence in criminal proceedings raised the question: are legal judgments influenced by neuroscientific evidence? Scientifically, this question was also motivated by a set of studies suggesting that neuroscientific information is seductively alluring in nonlegal contexts (Weisberg et al., 2008, 2015). It was further motivated by initial studies suggesting that neuroimages are uniquely persuasive (i.e., McCabe & Castel, 2008), but these effects have not been replicated (Gruber & Dickerson, 2012; Hook & Farah, 2013; Michael et al., 2013).

Although the experimental work on criminal cases reviewed above is methodologically varied, taken together it suggests that some legal judgments are influenced by neuroscientific evidence (for summary, see Table 1). Specifically, in all three studies that involved the death penalty, the presence of neuroscientific evidence (i.e., neuroscientific expert testimony, either alone or alongside neuroimages) decreased death sentences, at least for a subset of defendants (Appelbaum et al., 2015; Greene & Cahill, 2012; Saks et al., 2014). Similarly, in both studies that tested the effects of neuroscientific evidence on NGRI verdicts, such evidence reduced guilty verdicts (Gurley & Marcus, 2008; Schweitzer & Saks, 2011). However, across studies, neuroscientific evidence did not consistently lead mock jurors to return a not guilty verdict (Baker et al., 2013; Mowle et al., 2016 ; Schweitzer et al., 2011). Furthermore, with one exception (Allen et al., 2019), neuroscientific evidence did not reduce sentence length (Appelbaum et al., 2015; LaDuke et al., 2018; Mowle et al., 2016; Schweitzer et al., 2011). Interestingly, a study with real judges (who determine sentence lengths in real courtrooms) reported a mitigating effect of neuroscientific expert testimony on sentencing (Aspinwall, Brown, & Tabery, 2012).

Notably, while neuroscientific expert testimony influenced mock jurors in some studies with or without a neuroimage, neuroimages themselves had no effects above and beyond expert testimony (Greene & Cahill, 2012; LaDuke et al., 2018; Mowle et al., 2016; Saks et al., 2014; Schweitzer & Saks, 2011; Schweitzer et al., 2011). Furthermore, several studies found that no combination of neuroscientific testimony and neuroimages could persuade mock jurors to be more lenient (e.g., Baker et al., 2013; Mowle et al., 2016).

Furthermore, we asked whether there are factors moderating the efficacy of neuroscientific evidence on legal judgments. Several researchers tested whether psychiatric diagnoses may moderate these effects, but the results were inconsistent. Specifically, one study reported that neuroscientific evidence was more mitigating for defendants diagnosed with schizophrenia compared with those diagnosed with psychopathy (Saks et al., 2014), while another found equally mitigating effects for schizophrenia and psychopathy (Gurley & Marcus, 2008). A third study reported null effects for both diagnoses (Mowle et al., 2016). Similarly, results have been inconsistent across studies that tested whether the defendant’s future dangerousness is a moderator. Specifically, one study found that the effects of neuroscientific evidence on death sentences differed for defendants that were reported as having high versus low risk for being dangerous in the future (Greene & Cahill, 2012). In another study, defendants who were described as “treated”, and therefore low risk for future dangerousness, received lower prison sentences overall, although this did not moderate the effect of neuroscientific testimony (Allen et al., 2019).

Interestingly, although not tested directly as a moderator in any single study, the type of legal judgment appears to be a likely candidate. Indeed, across studies, neuroscientific evidence reduced death penalty sentences under most conditions (Appelbaum et al., 2015; Greene & Cahill, 2012; Saks et al., 2014), increased NGRI verdicts (Gurley & Marcus, 2008; Schweitzer & Saks, 2011), but did not increase non-NGRI not guilty verdicts (Mowle et al., 2016; Schweitzer et al., 2011) except in one study (Schweitzer et al., 2011). Furthermore, neuroscientific evidence did not influence length of prison sentences (Appelbaum et al., 2015; LaDuke et al., 2018; Mowle et al., 2016; Schweitzer et al., 2011) except in one study (Allen et al., 2019).

One explanation for this apparent effect of judgment type is that mock jurors evaluate evidence based on their adjudicative responsibility. For example, a mock juror tasked with choosing between a verdict of guilty and NGRI/GBMI (e.g., Gurley & Marcus, 2008) may be especially attuned to testimony regarding the defendant’s neural health. By contrast, a mock juror asked to recommend a sentence for a convicted defendant may not grant particular consideration to the defendant’s neural status when evaluating expert testimony.

Importantly, we must consider that multiple likely moderators have yet to be studied. For example, not a single study (to our knowledge) has varied the race of the defendant. This is a particularly important point when there is mounting evidence of racial bias in everyday judgments of various types (Pager, Bonikowski, & Western, 2009; Pletcher, Kertesz, Kohn, & Gonzales, 2008), whereby African-Americans are judged, for example, as less trustworthy (Stanley, Sokol-Hessner, Banaji, & Phelps, 2011) or more dangerous (Spector, 2001). Furthermore, there is evidence for racial bias in legal decisions specifically (Demuth, 2003; Hart, 2017; Hetey & Eberhardt, 2014; Johnson & Betsinger, 2009; Mitchell, Haw, Pfeifer, & Meissner, 2005; Mustard, 2001; Rachlinski, Johnson, Wistrich, & Guthrie, 2008; Sweeney & Haney, 1992). We might thus expect that the defendant’s race may be subject to bias, and may moderate the potential mitigating effect of neuroscientific evidence (e.g., such that African-American defendants will not be spared, even in cases in which white defendants will be). Other defendant-specific factors including age, gender, socioeconomic status, and physical attractiveness may also play roles in determining neuroscience’s efficacy in criminal trials (e.g., Ahola, Christianson, & Hellström, 2009; Doerner & Demuth, 2010; Freeman, 2006; Mustard, 2001; Walker & Woody, 2011).

On the other side of the courtroom, it is also possible that juror-specific factors may moderate the effects of neuroscientific evidence. For example, level of scientific training (and neuroscientific training in particular) is likely to moderate the degree to which neuroscientific information is mitigating. For instance, we predict that jurors (mock or real) who receive training on interpreting neuroscientific evidence, and/or determining its relevance, might respond differently to such evidence than those who have not received such training (Roskies, Schweitzer, & Saks, 2013). Although this has not been investigated directly, such findings would be consistent with those reported by Weisberg et al. (2008), whereby individuals with neuroscience expertise do not show the “seductive allure” effect. It is also possible that general attitudes about neuroscience may be influential, along with attitudes about mental illness, brain damage, free will, and personal responsibility. However, such factors have rarely been measured (c.f., Appelbaum et al., 2015).

Finally, trial-related factors, such as jury instructions, might also affect the jurors’ interpretation of evidence. While at least one influential study of the effect of jury instructions on the insanity defense found that mock jurors were insensitive to significant variation in instructions (Ogloff, 1991), this was not tested directly in any of the studies reviewed above. Thus, it remains possible that jury instructions do have an effect on jurors’ treatment of neuroscientific evidence.

We also sought to answer, *Why might neuroscience evidence be mitigating?* One explanation would be that such evidence is considered a “better argument” (i.e., more satisfying or more impactful), thus rendering the defense’s argument more satisfying or impactful. This explanation seems likely because, outside of the legal arena, it has been consistently reported that people find neuroscience explanations of psychological phenomena more satisfying (i.e., the “seductive allure effect”; Hopkins et al., 2016; Weisberg et al., 2018, 2008, 2015). Subsequent studies have suggested that this is due to a general preference for reductive explanations across the sciences (Hopkins et al., 2016; Weisberg et al., 2018). However, of the studies reviewed herein, none asked mock jurors whether neuroscientific evidence is more satisfying, and only two asked mock jurors whether they found such evidence persuasive (Gurley & Marcus, 2008; LaDuke et al., 2018). In one such study, neuroscientific evidence was associated with increased rates of NGRI verdicts, and those who rendered such a verdict reported finding such evidence more influential (Gurley & Marcus, 2008). However, the other study reported null effects (note that one additional study asked mock jurors such a question, but did not report results; Marshall et al., 2017). Thus, it remains unclear whether jurors broadly rate neuroscientific expert testimony as more satisfying or persuasive, and whether this might explain the mitigating effects found in some of the reviewed studies.

A powerful extension of this argument is that neuroscientific evidence specifically impacts jurors’ perceptions and cognitions regarding the defendant, including perception of responsibility, judgments of self-control, and predictions regarding future dangerousness. Indeed, in one study that tested this directly, the mitigating effect of neuroscientific evidence on verdicts was mediated by the amount of control the mock juror believed the defendant had over his actions (Schweitzer & Saks, 2011). Unfortunately, jurors’ perceptions and cognitions about the defendant remain a relative mystery, partly because such questions are not consistently asked (e.g., Gurley & Marcus, 2008; Mowle et al., 2016; Schweitzer et al., 2011) or because analyses of such questions are not consistently reported (e.g., Saks et al., 2014).

Nevertheless, the evidence is quite suggestive. For example, in three-quarters of the studies reported by Schweitzer et al. (2011), mock jurors were asked about their perception of the defendant’s self-control. In those studies, mock jurors who believed the defendant was more in control of his actions were more likely to render guilty verdicts and recommend longer sentences. Furthermore, a meta-analysis across these studies showed that all neuroscience conditions were associated with lower perceptions of control and responsibility (Schweitzer et al., 2011). Relatedly, Marshall et al. (2017) reported that neuroscience conditions were associated with lower perceptions of dangerousness, which were further related to lower sentences. Similarly, Appelbaum et al. (2015) reported that apprehension of the defendant (which includes perception of dangerousness) was related to sentence length. Finally, describing the defendant as having low versus high dangerousness has had a mitigating effect in two studies (Allen et al., 2019; Greene & Cahill, 2012).

Similarly, two studies found that the mitigating effect of neuroscientific evidence was related to decreased beliefs in the defendant’s self-control and other “deontological concerns” (e.g., moral responsibility, free will; Saks et al., 2014; Schweitzer & Saks, 2011). However, another study found that mock jurors’ perceptions of the defendant’s self-control were irrelevant to death sentence rates (Greene & Cahill, 2012). These conflicting results may suggest that the type of legal judgment, and thereby adjudicative responsibility, may play a role in moderating the cognitive mechanisms by which neuroscientific evidence persuades jurors towards leniency. Overall, although jurors’ perceptions and cognitions are a likely mechanism underlying the effect of neuroscientific evidence, to date not a single study has asked all the relevant questions and reported all the relevant analyses to address this hypothesis directly.

Our last question was, *Given the current state of the evidence, what might be productive avenues for future research?* As noted above, we strongly believe that additional research into moderating factors and cognitive mediators would benefit this field significantly. Specifically, we hope that moderators including the defendant’s race and gender will be tested as research has shown them to have an effect on legal judgments (e.g., Ahola et al., 2009; Demuth, 2003; Doerner & Demuth, 2010; Freeman, 2006; Johnson & Betsinger, 2009; Mitchell et al., 2005; Mustard, 2001; Rachlinski et al., 2008; Sweeney & Haney, 1992; Walker & Woody, 2011). In addition, we hope that future studies will probe juror cognitions about the evidence and about the experts delivering the evidence (i.e., whether they are persuasive, satisfying, and so on). Furthermore, we hope that such studies will also test the juror’s resulting beliefs about the defendant’s responsibility and character as possible cognitive mediators of any mitigating effects.

In addition, we believe that the type of criminal cases used could be varied. Indeed, the reviewed studies largely focus on murder and assault cases, in which the perceived costs of returning not guilty verdicts (or recommending lenient sentences) may be high. However, the majority of cases within the criminal justice system are not murder and assault cases, but rather lesser crimes. Edersheim, Brendel, and Price (2012) analyzed US court cases that introduced neuroscientific evidence as a *mens rea* defense. They found that the cases in which neuroscientific evidence successfully led to reduced charges or sentences were primarily property and drug crimes. Importantly, those are crimes where proof of greater intent is necessary for a guilty verdict, compared with violent crimes which do not share the same requirements of intent. Unfortunately, virtually no studies have examined the effects of neuroscientific evidence on property or drug crimes. Such studies could shed light on factors and circumstances that could affect the efficacy of neuroscientific evidence as it is used in courts today.

Finally, it is important to remember that neuroscience is already being used in criminal cases without regard to how well understood its effects are (Meixner, 2016). Therefore, while further research into its influence may not prevent (or promote) its use in criminal proceedings, additional research can help us educate judges and jurors about what neuroscientific evidence does (and does not) mean in a legal context. Indeed, some neuroscientists have specifically cautioned against overestimating the ability of neuroscience to answer questions of legal concern (Buckholtz & Faigman, 2014; Treadway & Buckholtz, 2011). This hesitation is due both to differences between the types of questions each field asks and answers, as well as the paucity of data linking neuroscientific findings to legally relevant aspects of criminal behavior. It is therefore ultimately possible that the greatest contribution of neuroscience to criminal justice will be its influence on how people think about free will, responsibility, and treatability in the context of criminal behavior, rather than to influence the legal decisions they make (Greene & Cohen, 2004).

## Data Availability

Not applicable.
